# The dynamics of educational transformation: Empirical insights into how innovation, collaboration, and professional development influence digital resource usage across 81 countries

**DOI:** 10.1371/journal.pone.0341914

**Published:** 2026-03-04

**Authors:** Liu Yuting, Rabiatul-adawiah Binti Ahmad Rashid, Zhu Xiaoying, Chen cheng

**Affiliations:** 1 School of Aviation Physical Education, Civil Aviation Flight University of China, Chengdu, Sichuan, China; 2 The School of Educational Studies, Universiti Sains Malaysia, Penang, Malaysia; 3 Zhongyuan Institute of Science and Technology, China; Zhejiang Normal University, CHINA

## Abstract

This study addresses a critical gap in understanding how teacher innovation drives the utilization of digital resources in education, mediated by teachers’ self-efficacy and collaboration, and moderated by professional development. Using data from 68,054 teachers across 81 countries in the PISA 2022 teacher questionnaire, we retained an analytic sample of 64,852 teachers after applying inclusion criteria and listwise deletion of missing values. We employed Partial Least Squares Structural Equation Modeling (PLS-SEM) to analyze these relationships. Results reveal that teacher innovation significantly predicts digital resource usage, with self-efficacy and collaboration serving as key mediators. Professional development amplifies these relationships. This study offers novel insights into the mechanisms linking teacher attributes to digital resource adoption, providing actionable strategies for policymakers to enhance educational digitalization. This study moves beyond the conventional individual-centered perspective of the technology acceptance model, offering a comprehensive and systematic strategy to support the digital transformation of education.

## 1. Introduction

Digitalization opens new possibilities for education. The digital transformation of education has been ongoing for years and saw a significant acceleration during the COVID-19 pandemic. The transformation introduces a fundamental shift in educational processes, incorporating technology not merely as a tool, but as a means to redefine teaching methods, learning experiences, and the overall educational landscape, thereby enhancing the potential for personalized education. The OECD Digital Education Outlook 2023 emphasized the diverse applications and impacts of digital technologies and educational resources [1]. Governments worldwide have made substantial investments in digital technology for schools to implement digital tools and resources. However persistent underutilization of digital resources despite infrastructure investments. Furthermore, debates on whether teachers and schools are fully utilizing the opportunities of digital technology in classrooms frequently lead to discussions about the technical infrastructure and the availability of digital tools in schools [2]. The use of digital resources within teacher-teaching activities is both an important a key measure to adapt to the digital transformation of education and an efficient approach to integrate digital resources into teaching skills to improve teaching quality and foster comprehensive student development. Sailer et al. (2021) proposed that rather than focusing on digital technology resources, teachers’ basic digital skills and technology-related teaching competencies are essential [3]. The focus should shift from equipping schools with technology to enhancing teachers’ abilities to effectively use these technologies.

Teacher plays a central role in digital transformation, as teachers guide students through the digital world, they will play a crucial role not only in fostering technological adoption but also in instilling the behaviors and values needed to manage the use of digital devices and thrive in an increasingly data-driven society [1]. Meanwhile, teachers are vital “change agents” in school digitalization processes and digital technologies may change the nature of their work [4]. According to the data released by PISA, even though there are 60% of teachers have participated in digital education training over the past year, around 20% of secondary education teachers report the need for further development. Teachers face significant challenges in mastering existing digital tools and resources tailored for education and utilizing the data collected by various digital systems to enhance the effectiveness and personalization of education for every student. Former studies explained teachers still lack adequate skills to fully leverage digital technologies in schools. This has sparked discussions about the specific skills and attributes teachers require to integrate digital devices and resources into their teaching and achieve high-quality instruction with technology.

Prior studies focus on individual factors (e.g., personal innovativeness, perceived usefulness, motivation, perceived ease of use, self-efficacy, and belief) and environmental factors (e.g., organizational climate, training policy) on teacher digital tools and ICT attitude and behavior [5–14] but neglect the interplay between innovation, collaboration, and institutional on teacher digital resource usage. Yao & Wang (2024) assessed the factors affecting preservice special education teachers’ intentions regarding the use of AI in education [14]. Drossel et al. (2017) examined school characteristics, teacher attitudes, teacher collaboration, and background factors to understand the frequency of computer use by secondary school teachers in the classroom [15]. Hava E (2023) pointed out that teachers’ self-innovativeness plays a critical role in distance learning teaching practices. However, research focusing on the precise mechanisms through which teachers’ innovation influences teachers’ usage behavior of digital resources in specific teaching activities remains limited [11].

Thus, this paper aims to investigate the relationship between teacher innovation and the usage behavior of digital resources in teaching activities, explore the mediating effect of self-efficacy, teacher collaboration, and the moderating effect of professional training related to digital resources on the relationship between teacher innovativeness and the usage behavior of digital resources in teaching activities. By integrating innovation diffusion theory and self-efficacy theory with teacher competency frameworks, this study not only advances the discourse on educational transformation but also offers actionable strategies for policymakers to accelerate the digitalization of education.

## 2. Literature review

### 2.1. Teacher innovation

Scholars have defined teacher innovation from different perspectives. Thurlings et al. (2015) believed that teacher innovation is an evolving process [16]; thus, they characterized teacher innovation as a process through which educators conceive, develop, apply, promote, and adjust new ideas to improve their job performance [17]. Described teacher innovation as the process of educators discovering and implementing new, effective teaching strategies aimed at fostering a student-centered learning environment that encourages creativity. This definition emphasizes not only the introduction of innovation but also the subsequent stages, including experimentation, implementation, refinement, and expansion.

As individuals become more innovative, they tend to view technology usage as more valuable, integrate it more effectively into their teaching, and develop a stronger interest in exploring the impact of technology use [8]. In a review of teacher innovation, Liu et al (2024) classify the factors influencing teacher innovation into three key categories: teacher characteristics and behaviors (such as lifelong learning, a survey thinking style, a proactive personality, multicultural experience, or a sense of humor, which tend to foster higher innovation levels), school characteristics (like leadership styles, professional support from colleagues and students, and a collaborative culture), and obstacles (including institutional contexts, educational policies, and national curriculum reforms). Meanwhile, they pointed out the outcomes of teacher innovation, including students’ intrinsic goal orientation, learning outcomes, and teachers’ instructional practices (such as technology acceptance and responsible teaching).

Teacher innovation is essential for improving educational processes and outcomes by integrating cross-disciplinary teaching skills, personalized and collaborative learning methods, problem-based learning strategies, increased cognitive engagement, and providing formative feedback to students [18]. Few studies examined the effect of teacher innovation on teacher usage behavior of digital resources. One similar study found that the relationship between teacher innovativeness and the intention to use technology was mediated by factors such as perceived ease of use, perceived usefulness, and subjective norms [8]. Sodergren et al (2023) discovered that innovations at the team level among teachers can boost their self-efficacy in areas such as teaching, classroom management, and student engagement [19]. while there is a lack of researches that focus on how teachers’ personal-level innovations affect teacher usage behavior of digital resources in teaching practice.

### 2.2. Teacher collaboration

Collaboration is defined as collective interaction among group members in all activities necessary to accomplish a shared task, with the understanding that it is not a fixed or uniform concept, but rather encompasses different types that vary in depth [20]. it can be viewed as an umbrella term, encompassing various collaborative concepts. Four distinct types of collaboration are positioned along a continuum from independence to interdependence, encompassing storytelling and idea scanning, providing aid and assistance, sharing, and engaging in joint work [21] indicated that collaboration involves partners working together on every aspect of the process, rather than dividing the work and later merging their individual contributions into the final outcome. Collaboration is regarded as distinct from collegiality, with the former referring to cooperative actions [22] while the latter focuses on the relationships among colleagues [23]. Kelchtermans (2006) described collegiality as relationships with colleagues grounded in mutual sympathy [22], and solidarity stemming from an equal work situation which aligns with the idea of Datnow’s (2011) distinction between collaborative cultures that foster spontaneous collaboration and contrived collegiality [24].

Studies indicate that teachers frequently limit collaboration to practical matters, such as discussing ideas and materials, planning lessons, determining the nature and content of assessments, and deciding on the pace and scope of instruction [20,25]. Providing teachers with opportunities to observe their peers, engage in discussions about their teaching practices, evaluate them, and reflect together can significantly improve teacher learning through collaboration [26]. Factors influencing teacher collaboration include communication, openness, participation, and a climate of trust, while hindering factors include teacher reluctance to share practices, lack of engagement, and teacher training [27]. Teacher collaboration is a continuum ranging from individual to strong team collaboration, offering benefits for students, teachers, and the school [20]. Studies have found that fostering a strong sense of collaborative school culture among teachers contributes to the promotion of collective teacher innovativeness through integrated professional learning activities. However, there are currently few studies on the impact of teacher innovation on teacher collaboration. According to Truijen et al. (2013), team innovation and the proactive tracking of educational developments are recognized as characteristics of effective teams, which, in turn, can enhance teacher collaboration [28]. Van Gasse et al (2017) argue that collaboration is the primary factor that explains teachers’ individual use of data [29]. Thus, the current study will investigate the role of teacher collaboration in the relationship between teacher innovation and digital resource use behavior, and examine how teacher innovation affects teacher usage behavior of digital resources through teacher collaboration.

### 2.3. Teacher self-efficacy

Self-efficacy refers to the belief in one’s ability to plan, organize, and carry out the actions necessary to achieve specific goals [30]. It is extensively utilized in education, psychology, and sociology, impacting teacher achievement, instructional practices, psychological well-being, behavioral modifications, social engagement, and career progression. Tschannen-Moran & Hoy (2001) described teacher self-efficacy as the belief in one’s capacity to deliver effective instruction, maintain classroom discipline, and foster student motivation for learning [31].

Teachers who possess high self-efficacy tend to embrace innovative teaching techniques, handle classroom management more effectively, and demonstrate increased confidence when confronting teaching challenges [32]. Prior studies have established a connection between self-efficacy beliefs, particularly technology self-efficacy, and the use of digital technology in teaching [14]. However, there is a lack of empirical evidence to confirm the association between teacher innovation and general self-efficacy. Specifically, limited research has explored the mediating role of teacher self-efficacy in the relationship between teacher innovation and the behavior of using digital resources. One exception study revealed that the self-efficacy of pre-service special education teachers is positively linked to their intention to use AI in education [14]. In this research, self-efficacy is conceptualized as a teacher’s confidence in their ability to manage the classroom, deliver effective instruction, and foster student engagement. Our study will explore the association between teacher innovation, teacher self-efficacy, and the usage behavior of digital resources, and examine whether teacher self-efficacy mediates the association between teacher innovation and the usage behavior of digital resources.

### 2.4. Professional development

Teacher professional development is understood as the process through which teachers acquire new knowledge, enhance their learning skills, and apply this learning in their practice to improve student outcomes [33], and it also encompasses educational experiences that are directly related to a teacher’s professional role, with the goal of acquiring and applying new knowledge and skills to enhance performance and ultimately improve student achievement[34]. Teachers who take an active role in their professional development are more capable of adapting and incorporating changes into their teaching methods [35]. Teacher professional development is shaped by personal and organizational factors, collaborative efforts among teachers, a supportive school environment, and engagement with external resource experts [33]. Recent work on STEM education has highlighted that teacher professional development is a critical lever for system-level readiness and instructional quality [36]. Similarly, intensive cognitive and strategy-focused training programmes, such as SMART, have been shown to enhance students’ higher-order thinking and decision-making skills [37], underscoring the broader potential of well-designed professional learning and training interventions to transform educational practices.

Teacher digital training is essential as it not only demonstrates how to utilize available tools but also provides clear, practical insights into their relevance and intended purposes. Programmed professional development in ICT integration for teacher educators led to significant progress, with technological pedagogical knowledge and participation in a community of practice emerging as key factors [38]. Previous studies revealed that longer hours of ICT training and the school ICT training plans boost teacher digital educational resources [13] while insufficient teacher training and limited ICT training focused on digital competence and professional development pose significant challenges [39]. Teacher participation in high-quality professional training equips them with the ability to acquire the essential tools and teaching competencies, which helps to transform intentional behaviors into actual usage of digital educational resources in teaching practice [40]. Nevertheless, there has been limited focus on exploring whether professional training in the use of digital resources influences the relationship between teacher innovation and the usage behavior of digital resources.

### 2.5. Teacher usage behavior of digital resources

Digital resources contain static, taking the form of digital textbooks, audio and video content, courses, or articles; or dynamic, leveraging interactive features and adaptive content to provide more personalized learning in interactive learning environments (quizzes, games, simulations, etc.) [1]. Digital resources provide possibilities for instructional design in the following three areas: individualized instruction, inquiry-based learning, and situated learning. Resources designed for individualized instruction will encourage self-paced, independent learning while supporting social learning activities to assess student comprehension. On the other hand, resources focused on situated cognition will prioritize the adaptability of content and the transfer of knowledge across various contexts or knowledge domains [41].

Teacher usage behavior of digital resources is conceptualized as teacher receptivity, adoption, and implementation of digital resources and their continual exploration of integrating digital tools and technologies into their teaching practices [42]. Teacher usage behavior of digital resources is influenced by a combination of internal and external factors. First, the Technology Acceptance Model (TAM) proposed that teacher usage behavior in digital resources could be influenced by factors such as perceived usefulness, perceived ease of use, subjective norms, and attitudes toward usage [43]. Yao & Wang (2024) demonstrated the ease of use and perceived usefulness of digital resources in education impact preservice special education teacher intention to use digital resources [14]. Secondly, theoretically, there are some arguments teachers with higher levels of belief, teaching experience, expertise, and skills, who participate in professional development and have improved facilitating conditions, are more likely to use digital resources regularly [5,13,40,44,45]. Teacher innovation and self-efficacy also could affect this behavior [9,46]. Thirdly, Empirical studies have demonstrated that school climate factors, including collaboration and social support among teachers and school leaders, play a key role in enabling teachers’ usage behavior of digital resources [15,47].

In summary, the literature has identified innovativeness, self-efficacy, collaboration, and professional learning as key factors that can influence teacher usage behavior of digital resources. Building on the above, our research questions were:

Q1: What is the association between teacher innovation and the teacher usage behavior of digital resources in teaching practices?

Q2: What is the association between teacher innovation, teacher self-efficacy, and the teacher usage behavior of digital resources in teaching practices?

Q3: What is the association between teacher innovation, teacher collaboration, and the teacher usage behavior of digital resources in teaching practices?

Q4: Does teacher participation in professional development related to digital resources impact the relationship between teacher innovation and the teacher usage behavior of digital resources in teaching practices?

## 3. Theoretical basis and research hypotheses

### 3.1. Theoretical framework

The diffusion of innovations theory is a hypothesis outlining how new technological and other advancements spread throughout societies and cultures, from introduction to widespread adoption. The diffusion of innovations theory seeks to explain how and why new ideas and practices are adopted. The diffusion of innovations theory has been used include agriculture, social work, communication, and criminal justice [48]. Building on Rogers’ (2003) Diffusion of Innovation Theory, this study frames teacher innovativeness as the hallmark of “early adopters,” positions collaboration as “re-invention within the social system,” and views professional development as a key component of the “implementation stage” supported by institutional structures [49]. Additionally, Self-efficacy theory (SET), a component of Bandura’s (1986) social cognitive theory, posits that behavior is primarily influenced by two key factors: perceived self-efficacy and outcome expectancies. It has significantly advanced our understanding of human motivation. Research highlights self-efficacy as a core internal driver shaped by both personal and environmental factors, which in turn affects key motivational outcomes such as decision-making, effort, persistence, and achievement. Its application across various contexts has also led to adaptations of the original theory to better align with diverse settings. Bandura’s (1977) Self-Efficacy Theory elucidates how teachers’ confidence mediates the link between innovation and behavioral adoption [30]. By integrating these perspectives, this framework moves beyond the traditional, individual-focused Technology Acceptance Model (TAM), offering a comprehensive explanation for the digital transformation of education.

Furthermore, the proposed framework is designed to be exploratory, aims to discover and explain how innovation, collaboration, and self-efficacy interact to shape teachers’ digital resource usage across 81 countries. Guided by the Diffusion of Innovation and Social Cognitive perspectives, the model seeks to uncover potential global mechanisms rather than to confirm predetermined causal structures. This exploratory orientation allows the research to generate new theoretical insights that can inform future confirmatory investigations.

### 3.2. Conceptual framework

**Fig 1:**
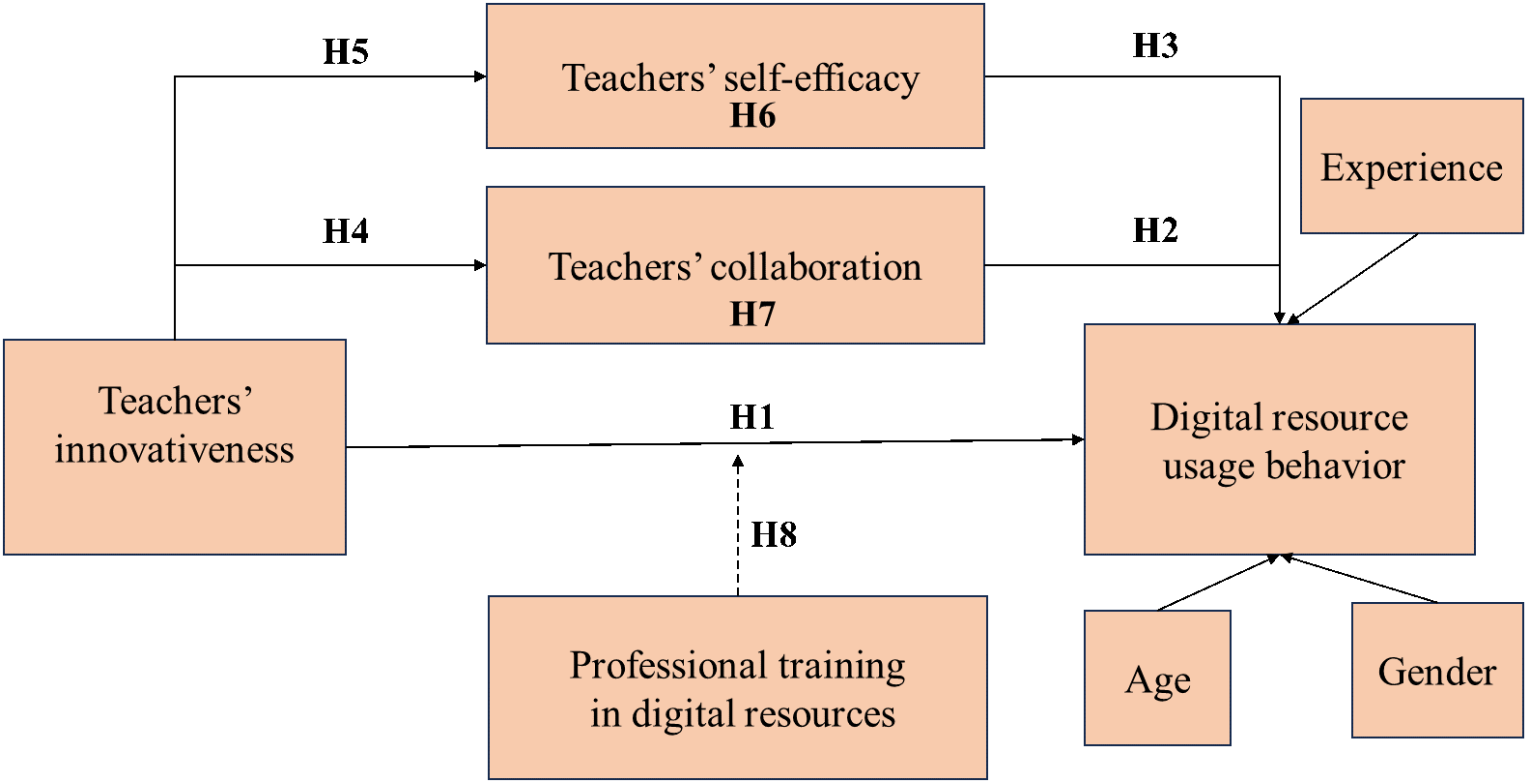
Conceptual model.

The proposed conceptual model in this study is informed by the Diffusion of Innovations Theory [49], which is used to explain how innovations (new technologies, new products, new ideas, etc.) The process of diffusion and acceptance in a social system. The theory attempts to understand and explain how an innovation spreads, how quickly it spreads, and how much it is adopted. Drawing from the literature discussed above, the conceptual framework for the current study is developed. Fig 1 illustrates the relationships among the key variables explored in the article, including teacher innovation, teacher self-efficacy, teacher collaboration, teacher professional development, and teacher usage behavior of digital resources.

The conceptual model presented in this article proposes teacher innovation as the independent variable, with teacher usage behavior of digital resources serving as the dependent variable. Teacher self-efficacy and teacher collaboration as a mediating variable. Teacher professional development as a moderating variable (Fig 1).

### 3.3. Research hypothesis

Prior studies indicate that innovative teachers are more likely to adopt digital tools in teaching [8,11]. Innovation drives experimentation with new methodologies, making digital resource integration a natural extension of creative pedagogy. Thus, H1: Teacher innovation is significantly associated with teacher usage behavior of digital resources.

Collaboration facilitates knowledge-sharing about digital tools [20,29]. Teachers who jointly develop instructional strategies are more likely to leverage digital resources effectively. Thus, H2: Teacher collaboration is significantly associated with teacher usage behavior of digital resources.

Self-efficacious teachers exhibit greater confidence in using technology [14,30]. This confidence directly translates to increased digital resource utilization. Thus, H3: Teacher self-efficacy is significantly associated with teacher usage behavior of digital resources.

Innovative teachers seek collaborative environments to refine ideas [19,28]. Innovation thus acts as a catalyst for professional teamwork. So, H4: Teacher innovation is significantly associated with teacher collaboration.

Innovation reinforces teachers’ belief in their capabilities [32]. Successful implementation of novel approaches boosts confidence in managing digital tools.

Thus, H5: Teacher innovation is significantly associated with teacher self-efficacy.

As innovation enhances self-efficacy, which in turn increases digital resource adoption [9,42]. Thus, H6: Teacher self-efficacy mediates the association between teacher innovation and digital resource usage.

Collaboration is expected to channel innovative ideas into practical digital applications [15,27]. Thus, H7: Teacher collaboration mediates the association between teacher innovation and digital resource usage.

Training amplifies innovation’s impact by equipping teachers with technical skills [13,37]. Thus, H8: Professional development moderates the relationship between teacher innovation and digital resource usage.

## 4. Design and method

### 4.1. Data and sample

The data are sourced from the PISA 2022 dataset, which includes comprehensive responses from individual students, school principals, teachers, and parents, and were selected for their global representativeness and rigorous sampling, enabling cross-national comparisons (OECD, 2023). We used the computer-based teacher questionnaire for the PISA 2022 file (CY8_202111_QST_FT_TCQ_NoNotes.docx). The dataset can be accessed at https://www.oecd.org/en/data/datasets/pisa-2022-database.html. In total, the teacher questionnaire dataset included 68,054 teachers. To ensure representativeness, teacher sampling weights (W_TCHWT) were applied in all descriptive statistics and percentage calculations. The analytic sample for the structural equation modelling was restricted to teachers whocompleted the full teacher questionnaire, reported at least one year of teaching experience, and had no missing data on the focal constructs and control variables. After applying these criteria and listwise deletion of missing values, 64,852 teachers were retained in the analytic sample, comprising 38,655 females (59.6%) and 26,197 males (40.4%), from public and private schools in 81 countries. The data were collected cross-sectionally from October 2021 to April 2022 (OECD.org) [50]. The basic information of the sample as Table 1.

**Table 1:** Basic information of the sample (N = 68054).

Category		Frequency	Percentage (%)
Gender	Total	68054	
	Female	38655	56.8
	Male	26197	38.5
	Missing	3202	4.7
Age	Total	68054	100
	20-30	8990	13.2
	31-40	17664	26
	41-50	22385	32.9
	51-60	13926	20.5
	61-70	4178	6.1
	Missing	911	1.3
Teaching Experience	Total	68054	100
	0-10	22324	32.8
	11-20	22211	32.6
	21-30	14923	21.9
	31-40	6424	9.4
	41-50	1030	1.5
	Missing	1142	1.7
OECD Country	Total	68054	100.0
	No	40834	60.0
	Yes	27220	40.0

### 4.2. Measures

In the conceptual model of this article, the dependent variable was labeled as teacher usage behavior of digital resources. This latent variable is defined by nine indicators (TC220Q02JA, TC220Q04JA, TC220Q06JA, TC220Q07JA, TC220Q08JA, TC220Q09JA, TC220Q10JA, TC220Q11JA, TC220Q12JA) from the original PISA scale of “Usage digital resources in teaching activities”. It measured the frequency of teacher usage of digital resources in different processes of teaching activities [50]. The core question was “This school year, how often did you do the following activities? Cronbach’s alpha = 0.905”. All items were answered by choosing one of the following choices: “ever or almost never, about once or twice a year, about once or twice a month, about once or twice a week, every day or almost every day.”

The mediating variables are two variables, the first one is labeled as teacher self-efficacy, which was assessed using 12 items (TC199Q01HA to TC199Q012HA) on a 4-point scale (e.g., “Provide an alternative explanation when students are confused”), with a Cronbach’s alpha of 0.921. This scale comprises three subscales: self-efficacy in classroom management, instruction, and student engagement. The second one is teacher collaboration, which was assessed using four items (TC046Q04NA to TC046Q07NA, e.g., Work with other teachers in my school to ensure common standards in evaluations for assessing student progress, Cronbach’s alpha = 0.796).

The moderating variable is a binary variable, which is labeled according to information about teacher professional development activities related to digital resources that occurred within the 12 months preceding data collection (TC020Q11NA to TC020Q14NA) (e.g., “During the last 12 months, did you participate in any of the following activities?”) Ten items (TC020Q1NA to TC020Q10NA) measured general learning activities, and the last four items (TC020Q11NA to TC020Q14NA) specifically measured the professional development activities of digital resources. For each of the 14 items, participants were asked to select either “yes” or “no” to indicate their experience. In this study, the authors only focus on the professional development of digital resources; thus, the authors adapted adapt the last four items (TC020Q11NA to TC020Q14NA). This variable was coded as 0/1 to represent whether a teacher participated in at least one of the four professional learning activities related to digital resources in the past 12 months.

The Independent variable is teacher innovativeness measured on nine items (TC234Q01JA to TC234Q09JA). These items measured the extent to which teacher’ self-perception and attitude toward their creative thinking. All items were responded to using a four-point Likert-type scale, ranging from “strongly disagree” to “strongly agree.” The main question was “To what extent do you agree or disagree with the following statements? Cronbach’s alpha = 0.85.”

All items were responded to using a four-point Likert-type scale, ranging from “strongly disagree” to “strongly agree.” The main question was, “To what extent do you agree or disagree with the following statements?”

To account for potentially confounding effects, we added demographic control variables from the PISA 2022 teacher questionnaire, including gender, age, and teaching experience.

a) gender (TC001Q01NA, coded: 1: female, 2: male),
b) age (TC002Q01NA, metric),
c) teaching experience (TC007Q02NA, metric).

### 4.3. Data analysis

The research model proposed in this study was tested through the Partial Least Squares path modeling technique using the Smart-PLS version 4.0 due to several reasons; (a) Model Complexity; The research involves multiple variables and their relationships, including teacher innovation, teacher self-efficacy, teacher collaboration, teacher professional development, and the teacher usage behavior of digital resources. PLS-SEM is particularly effective for managing complex models that include multiple causal relationships, mediation, and moderation effects. (b) Prediction Orientation: PLS-SEM is mainly used for predictive modeling, emphasizing the explanation and prediction of relationships between variables. This is aligned with the study’s objective to understand and predict the impact of teacher innovation on the teacher usage behavior of digital resources, to explore the mediating roles of teacher self-efficacy, and teacher collaboration, and to explore the moderating role of teacher professional development on related variables, moderation by professional development was examined using partial least squares multi-group analysis (PLS-MGA), comparing structural paths between teachers who did and did not participate in digital-resource-related professional development. PLS-MGA allows comparing path coefficients between groups in a non-parametric way and is well suited for large, complex models. The measurement model evaluates the proposed relationships between indicators and latent variables, while the structural model assesses the proposed pathways between exogenous (independent) and endogenous (dependent) latent variables. Furthermore, Because the data were collected using self-reported measures at a single point in time, the authors assessed potential common method bias (CMB). A full collinearity test was conducted by examining the variance inflation factor (VIF) values of all latent constructs. All VIF values were well below the recommended threshold of 3.30, suggesting that multicollinearity and CMB were not serious concerns in this study [51,52]. In addition, discriminant validity was supported by both the Fornell–Larcker criterion and HTMT analysis, further mitigating the risk of CMB.

The dataset exhibited less than 5% missingness per variable and was confirmed to be missing completely at random (MCAR). Listwise deletion was applied. Robustness of the model was further verified using weighted samples, which produced consistent path directions and significance levels.

## 5. Result

### 5.1. Reliability and validity

#### 5.1.1. Measurement model assessment.

To evaluate reliability and validity with PLS, researchers commonly compute a set of indicator composite reliabilities (CR), individual item reliability, Cronbach’s alpha, and average variance extracted (AVE) [53,54].

*Individual item reliability (Loadings):* It is recommended to use the outer loadings of each item for all constructs to assess individual item reliability [55]. The threshold is bigger than 0.70 means that the individual items have good reliability. In this study, as shown in Table 2, nearly all item reliabilities exceed 0.7, indicating that the study satisfies the criteria for individual item reliability.

**Table 2:** Measurement Model.

Construct	Items	Loading	Weights	P-value	Cronbach’s alpha	Composite reliability (CR)	Average variance extracted (AVE)
**/Code**
(TCL)	Teacher Collaboration						
TC046	On average, how often do you do the following in this school?				0.796	0.803	0.623
TC046Q04NA		0.763	0.288	0.000			
TC046Q05NA	Engage in discussions about the learning development of specific students	0.814	0.325	0.000			
TC046Q06NA	Work with other teachers in my school to ensure common standards in evaluations for assessing student progress	0.852	0.35	0.000			
TC046Q07NA	Attend team conferences	0.721	0.302	0.000			
(TIN)	Teacher Innovation						
TC234	To what extent do you agree or disagree with the following statements?				0.85	0.856	0.528
TC234Q01JA	I am very creative.	0.799	0.218	0.000			
TC234Q02JA	I enjoy projects that require creative solutions.	0.829	0.223	0.000			
TC234Q03JA	I enjoy solving complex problems.	0.661	0.213	0.000			
TC234Q04JA	I enjoy learning new things.	0.688	0.212	0.000			
TC234Q05JA	I enjoy artistic activities.	0.703	0.154	0.000			
TC234Q06JA	I express myself through art.	0.655	0.155	0.000			
TC234Q08JA	I have difficulty using my imagination.	0.731	0.198	0.000			
(TSEF)	Teacher self-efficacy				0.921	0.926	0.534
TC199	In your teaching, to what extent can you do the following						
TC199Q01HA	Get students to believe they can do well in schoolwork	0.731	0.112	0.000			
TC199Q02HA	Help my students value learning	0.761	0.109	0.000			
TC199Q03HA	Craft good questions for students	0.735	0.12	0.000			
TC199Q04HA	Control disruptive behavior in the classroom	0.684	0.085	0.000			
TC199Q05HA	Motivate students who show low interest in school work	0.736	0.106	0.000			
TC199Q06HA	Make my expectations about student behavior clear	0.703	0.115	0.000			
TC199Q07HA	Help students think critically	0.752	0.128	0.000			
TC199Q08HA	Get students to follow classroom rules	0.733	0.093	0.000			
TC199Q09HA	Clam a student who is disruptive or noisy	0.694	0.088	0.000			
TC199Q10HA	Use a variety of assessment strategies	0.743	0.138	0.000			
TC199Q11HA	Provide an alternative explanation, for example, when students are confused	0.723	0.12	0.000			
TC199Q12HA	Implement alternative instructional strategies in my classroom	0.766	0.15	0.000			
(UBDR)	Teacher usage behavior of digital resources						
TC220	This school year, how often did you do the following activities?				0.905	0.908	0.570
TC220Q02JA	Use digital resources to design tasks	0.725	0.132	0.000			
TC220Q04JA	Use digital resources to explore new teaching methods	0.772	0.147	0.000			
TC220Q06JA	Use digital resources to enable student collaboration	0.82	0.158	0.000			
TC220Q07JA	Use digital resources to provide feedback to students	0.819	0.151	0.000			
TC220Q08JA	Use digital resources to provide access to instructional material for students who cannot physically attend class	0.756	0.141	0.000			
TC220Q09JA	Use digital resources to communicate with parents or guardians	0.691	0.146	0.000			
TC220Q10JA	Use online tools or computer-based testing to assess student learning	0.74	0.131	0.000			
TC220Q11JA	Use digital resources to share ideas or resources with colleagues	0.788	0.178	0.000			
TC220Q12JA	Take part in professional communities of practice online	0.668	0.139	0.000			

*Note: TCL: Teacher Collaboration; TIN: Teacher Innovation; TSEF: Teacher self-efficacy; UBDR: Teacher usage behavior of digital resources.*

*Cronbach alpha (CA):* Similar to Cronbach’s alpha, which measures internal consistency reliability, a composite reliability of 0.70 or higher is deemed acceptable for research purposes [56]. The Cronbach alpha in the present study achieved the rule of thumb for Cronbach alpha values criteria, as depicted in Table 2.

*Composite reliability (CR):* CR represents internal consistency reliability, with researchers setting the threshold at 0.7 or higher. Table 2 displays every item’s composite reliability in the current study, indicating that all constructs were found to have adequate internal consistency.

*Average Variance Extracted (AVE):* The AVE measures the proportion of variance captured by the indicators about measurement error [56], with a recommended threshold of greater than 0.50 to justify the use of a construct [53]. In this study, the AVE for all constructs meets the minimum threshold of 0.50, ensuring sufficient convergent validity (see Table 2).

*Discriminant Validity (DV):* This study analyzed the correlation between constructs and factor loadings to assess discriminant and convergent validity. According to Fornell & Larcker (1981), discriminant validity is demonstrated when a latent variable explains more variance in its associated indicator variables than it shares with other constructs in the model. Discriminant validity is established when the square root of each construct’s AVE is greater than the correlation between the construct and other latent variables. Table 3 shows the results of the Fornell-Lacker Criterion. Henseler et al. (2015) introduced a new criterion for evaluating discriminant validity in variance-based structural equation modeling [57]. They recommend the HTMT criteria of less than 0.85. Table 3 displays the results of HTMT, except for the second-order; other results are below 0.85. The measures used in this study demonstrated an adequate level of discriminant validity.

**Table 3:** Discrimination validity results.

Fornell–Larcker Criterion Results					Heterotrait-Monotrait ratio (HTMT) Results				
	TCL	TIN	TSEF	UBDR		TCL	TIN	TSEF	UBDR
TCL	0.789				TCL				
TIN	0.195	**0.726**			TIN	0.228			
TSEF	0.212	0.369	**0.731**		TSEF	0.237	0.404		
UBDR	0.448	0.298	0.291	**0.755**	UBDR	0.523	0.334	0.309	

#### 5.1.2. Assessment of structural model.

**Fig 2:**
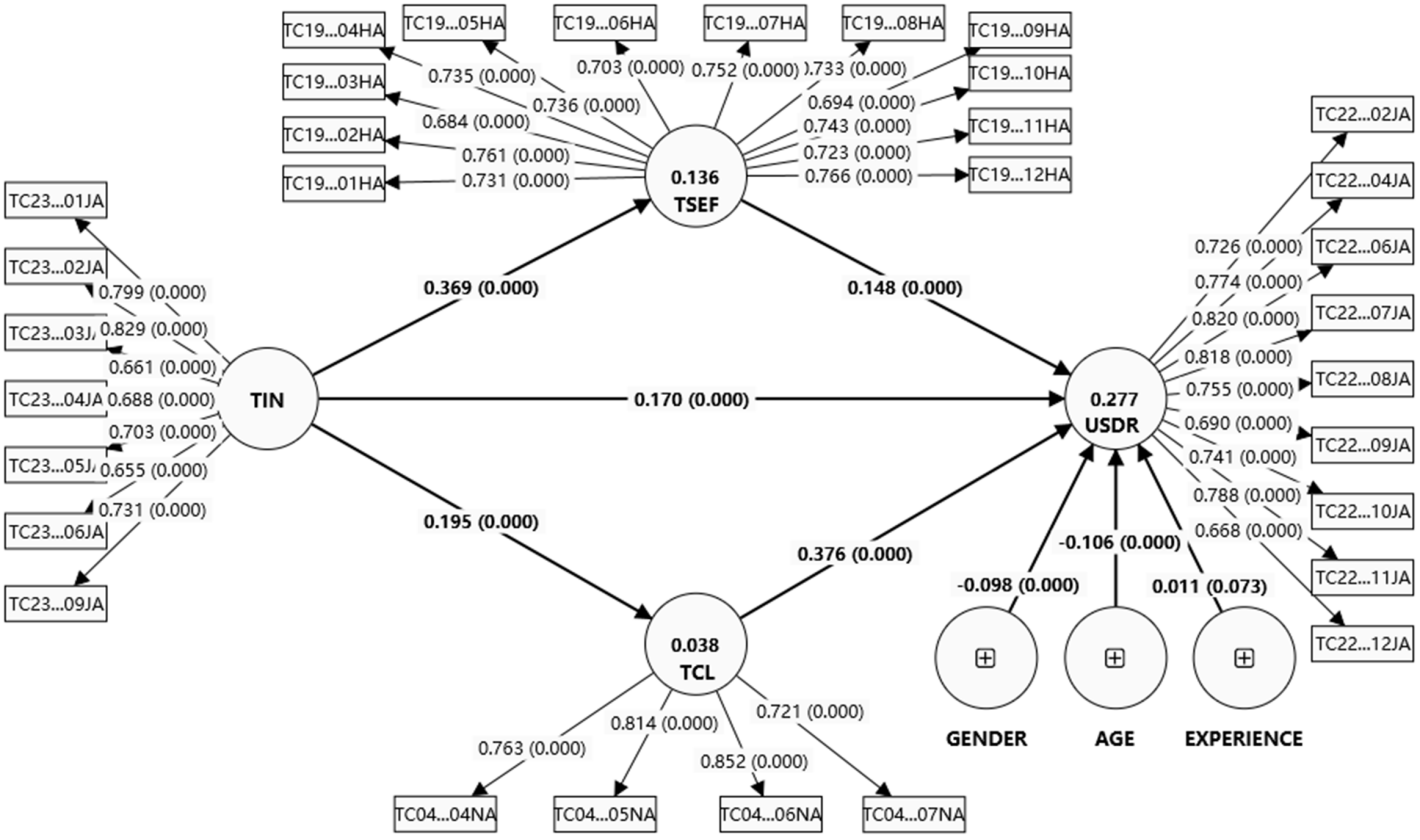
Bootstrapping result (Path coefficients and p-value).

According to Chin [54,58], the bootstrapping procedure could generate t-statistics and standard errors, and the R² value could explain the proportion of variance. Therefore, to evaluate the full model, R^2^ values were used to indicate the explanatory power of the model. Effect sizes (f^2^) and predictive relevance (Q^2^) were used to assess the model’s predictive capabilities. The bootstrapping was also used to obtain confidence intervals for the path coefficients and to test indirect effects. The standardized root mean square residual (SRMR) was utilized to assess the model fit. The results of the structural model are shown in Fig 2.

*Coefficient of determination (R²)*: In PLS-SEM, R^2^ values of 0.67, 0.33, and 0.19 are interpreted as substantial, moderate, and weak, respectively(Chin, 1998). In this study, the value of R^2^ is shown in Table 4.

**Table 4:** Structural model results.

Construct/Paths	R-square	Q² (=1-SSE/SSO)	f-square	VIF	SRMR
TCL	0.038	0.023			
TSEF	0.136	0.07			
UBDR	0.265	0.149			
TCL → UBDR			0.189	1.073	
TIN → TCL			0.039	1.000	
TIN → TSEF			0.158	1.000	
TIN → UBDR			0.033	1.184	
TSEF → UBDR			0.025	1.189	
Model-fit					0.053

*The Predictive Relevance (Q*^*2*^*):* In this study, cross-validated redundancy (Q^2^) was used to assess the effects of latent variables [59]. A Q^2^ value greater than zero indicates the presence of predictive relevance in the model [60]. The Q^2^ values for the current study can be found in Table 4 and are above zero. This suggests that the model has predictive validity.

*The effect sizes(f²):* The effect sizes (f^2^) measure the magnitude of the effect of an independent variable on a dependent variable in a structural model. In PLS-SEM, the F^2^ values are interpreted as follows: f^2^ > 0.35 indicates a large effect size, 0.15 < f^2^ < 0.35 indicates a medium effect size, and f^2^ < 0.02 indicates a small effect size(Chin, 1998). The findings of this study indicate that all f^2^ values exceed 0.02. (see Table 4).

*Structural model collinearity issue:* The VIF values for all variables were utilized to assess the presence of collinearity in the structural model. As noted by scholars, a VIF value of 3.30 or below is generally considered to be free from bias [59]. All full collinearity VIF values were below the recommended threshold of 3.30(refer to Table 4), suggesting that multicollinearity and common-method variance are unlikely to pose severe threats to the estimated relationships. Nevertheless, because all variables were measured by self-report in a single questionnaire, common-method bias cannot be completely ruled out and the results should be interpreted with appropriate caution [61].

*Model fit:* SRMR is used to measure the model fit of the construction. As scholars suggested the value of SRMR lower than 0.08 is considered to satisfy the model fit [60]. The SRMR value for the current study is 0.053 (see Table 4), lower than 0.08, indicating that the model demonstrates a good fit.

*Measurement invariance:* In this study, multi-group analysis (MGA) was conducted using partial least squares path modeling through Smart-PLS 4.0 software. Once the groups have been identified, the MGA enables researchers to assess disparities among them in two comparable models [62]. Before evaluating the multiple group analysis (MGA), we first conducted a measurement invariance test on the composite model (MICOM) to compare the impact of teachers’ participation in digital resource-related professional development activities on the relationship between teacher innovation and teachers’ digital resource use behavior. The MICOM test followed three steps (Table 5). We then performed a permutation multiple group analysis and a bootstrap multiple group analysis procedure. The results are shown in Table 5.

**Table 5:** Results of invariance measurement testing using permutation.

Constructs	Configurational Invariance (step 1)		Compositional Invariance (step 2)	Partial Measurement Invariance	Equal Mean Assessment (step3a)		Equal Variance Assessment (step3b)		Full Measurement Invariance
		Original Correlation	5%		Original Differences	confidence interval	Original Differences	confidence interval	
TIN	yes	0.999	1	yes	0.29	[-0.019-0.019]	-0.022	[-0.026-0.026]	no/yes
USDR	yes	1	1	yes	0.702	[-0.018-0.018]	-0.095	[-0.021-0.022]	no/no

*Mediation and moderation analysis:* According to Baron & Kenny (1986), the key feature of an indirect effect is the involvement of a third variable that acts as an intermediary in the link between the dependent and independent variables [63]. In contrast, a moderator is a variable—either qualitative or quantitative—that influences the direction and/or strength of the relationship between an independent or predictor variable and a dependent or outcome variable. According to Chin (2010), when testing the mediating effect, the authors should examine the total indirect effect first [64]. if the result is statistically significant means that the third variable acts as an intermediary in the relationship between dependent and independent variables. We used non-parametric bootstrapping with 5,000 resamples to obtain standard errors, t-statistics, and two-tailed 95% confidence intervals for all path coefficients and indirect effects. The results (Table 6) (Fig 2) show that teacher innovation, teacher collaboration, and teacher self-efficacy have proven a direct, statistically significant, positive association with teacher usage behavior of digital resources (H1: t = 79.594, p < .005, H2: t = 106.26, p < .005, H3: t = 38.712, p < .005). Teachers who have a high personal perception of their innovation, a high self-efficacy, and are more likely to collaborate with others will more frequently use digital resources in their teaching practice. Thus, supporting Hypotheses 1, 2, and 3. Teacher innovation has proven a direct, statistically significant, positive association with teacher collaboration and teacher self-efficacy (H4: t = 49.148, p < .005, H5: t = 105.355, p < .005). the results mean that the more innovative teachers are in their teaching, the more likely they are to collaborate effectively with other teachers and the more confident they are in their teaching abilities and outcomes. Hypotheses 4 and 5 are supported.

**Table 6:** Path coefficients and hypothesis testing.

Path relationship	Hypothesis	Original sample (O)	Sample mean (M)	Standard deviation (STDEV)	T statistics (|O/STDEV|)	P values	Decision
Directly effect							
TCL → UBDR	H2	0.384	0.384	0.004	106.26	0.000	Supported
TIN → TCL	H4	0.195	0.195	0.004	49.148	00.00	Supported
TIN → TSEF	H5	0.369	0.369	0.004	105.355	0.000	Supported
TIN → UBDR	H1	0.298	0.298	0.004	79.594	0.000	Supported
TSEF → UBDR	H3	0.147	0.147	0.004	38.712	0.000	Supported
Indirect effect							
TIN → TSEF → UBDR	H6	0.054	0.054	0.001	36.566	0.000	Supported
TIN → TCL→ UBDR	H7	0.075	0.075	0.002	44.335	0.000	Supported
Total indirect effect							
TIN → UBDR		0.129	0.129	0.002	59.600	0.000	Supported

Teacher self-efficacy partially mediates the association between innovation and digital resource usage, indicating that innovation boosts self-efficacy, which in turn enhances usage behavior (TSEF H6, t = 36.566 ＞ 1.96, p ＜ 0.001). Collaboration serves as a significant mediator between innovation and digital resource usage, suggesting that innovation fosters collaboration, which subsequently increases usage behavior (TCL H7, t = 44.335 ＞ 1.96, p ＜ 0.001). These results support hypotheses H6 and H7.

**Fig 3:**
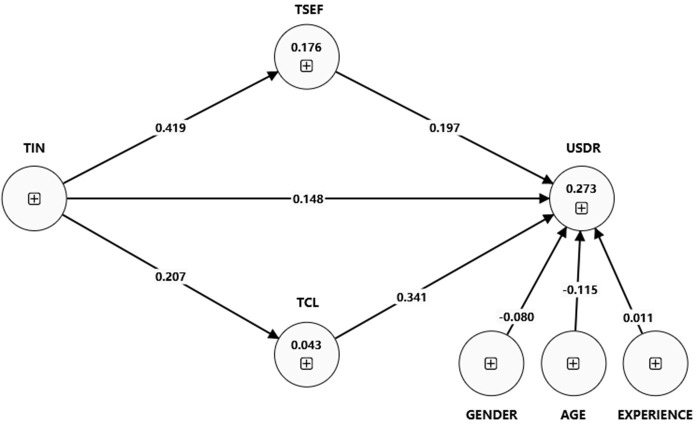
Structural model (pd-yes).

**Fig 4:**
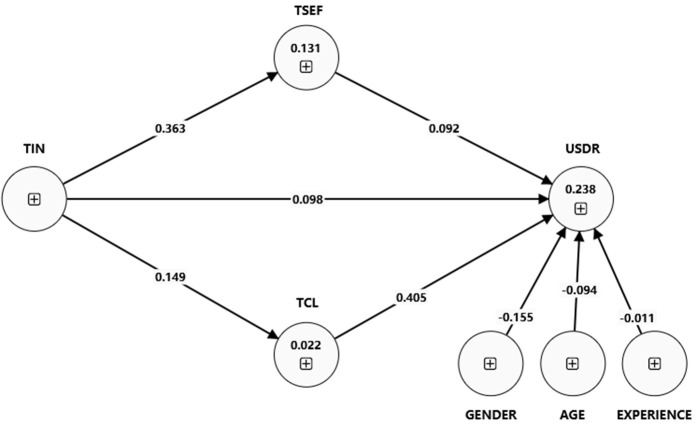
Structural model (pd-no).

As presented in Table 7, Fig 3, and Fig 4, the multigroup analysis reveals significant differences in the relationships between the variables for teachers who attended professional development and those who did not. The path coefficient for the direct effect of TIN on UBDR is 0.146 for attendees and 0.096 for non-attendees. The result supports hypothesis H8. The positive difference of 0.049 is statistically significant (p-value = 0), showing that TIN directly impacts UBDR more for attendees.

**Table 7:** Hypothesis results (PLS-MGA).

Path		Original	Original	confidence intervals		Difference	p-value
relationship	Hypothesis	(pd-yes)	(pd-no)	(pd-yes)	(pd-no)	(pd-yes vs pd-no)	(pd-yes vs pd-no)
TIN → UBDR	H8	0.146	0.096	[0.138-0.158]	[0.079-0.116]	0.049	0

Controlling variables on the dependent variable, the authors found that both age (β = -.0.106, p < .001) and gender (β = −.098, p < .001) were negatively associated with the teachers usage behavior of digital resources (Fig 2). While teachers’ experience shows no associated with teachers’ usage behavior of digital resources.

## 6. Discussion and Implications

The Results section presents four key findings of this article. Firstly, teacher innovation, teacher self-efficacy, and teacher collaboration were significantly related to teacher usage behavior of digital resources in teaching practices. Secondly, teacher innovation was significantly and directly associated with teacher self-efficacy and teacher collaboration. Thirdly, teacher self-efficacy and teacher collaboration play a partially indirect mediator role in the relationship between teacher innovation and teacher usage behavior of digital resources in teaching practices. Fourthly, the results demonstrated a statistically significant moderate effect of professional development of digital resources on the relationship between teacher innovation and teacher usage behavior of digital resources in teaching practices. Prior to exploring the key implications of these findings, it is essential to acknowledge some limitations and propose areas for further research to build upon this study.

### 6.1. Associate between teacher innovation and usage behavior of digital resources

This study supports the above suggestion that teacher innovation is positively associated with teacher usage behavior of digital resources in teaching practice. Teacher innovation enables the development and adoption of new teaching methods, making lessons more interactive and engaging, and can better meet the individual needs of students by designing activities that cater to different learning styles and abilities [18]. Teachers who demonstrate innovation are more inclined to adopt and incorporate digital tools into their teaching practices [8], through innovation, teachers can develop new and improved teaching methods that leverage digital resources. Innovative teachers use digital resources to create more dynamic and student-centered learning environments.

Teacher innovation plays a critical role in the effective application of digital resources in education. Innovative teachers adopt digital resources to develop and implement creative teaching strategies that enhance student engagement and learning outcomes. It’s crucial to encourage a culture of innovation among educators to unleash the full potential of digital resources in today’s educational settings.

### 6.2. Associate between teacher innovation, self-efficacy, teacher collaboration, and usage behavior of digital resources

The current finding reveals the connection between teacher innovation (TIN), self-efficacy (TSEF), teacher collaboration, and their usage behavior of digital resources (UBDR). The direct effect of TIN on TSEF (path coefficient: 0.369, T-statistic: 105.355), TSEF on UBDR (path coefficient: 0.147, T-statistic: 38.712), the direct effect of TIN on TCL (path coefficient: 0.195, T-statistic: 49.148), and TCL on UBDR (path coefficient: 0.384, T-statistic: 106.26) are significant. It has consistent results with the former research that teacher innovation is a crucial driver of both teacher team collaboration and teacher self-efficacy [11,19,28,65].

Additionally, the indirect effect of TIN and TCL on UBDR through TSEF (path coefficient: 0.054, T-statistic: 36.566) is also supported. The results indicate that innovative practices among teachers boost their self-efficacy, which in turn enhances their engagement with digital resources. Innovative teachers are more likely to collaborate, and this collaborative behavior subsequently also enhances their use of digital resources. Therefore, increasing teacher self-efficacy and promoting collaborative environments can amplify the positive impact of innovation on digital resource usage.

### 6.3. Impact of professional development in digital resources on the relationship between innovation and usage behavior

The direct effect of innovation on usage behavior (TIN – UBDR) is more pronounced for professional development (PD) related to digital resources attendees (path coefficients: 0.146) than for non-attendees (path coefficients: 0.096). This suggests that PD related to digital resources provides teachers with additional support and resources that enhance the direct and indirect pathways through which innovation influences the use of digital resources.

### 6.4. Limitations

The dataset of PISA2022 utilized in this study is derived solely from teachers’ self-reported perceptions and collected at a single point in time. Although statistical checks indicated that CMB is not a major threat in this study, the possibility cannot be fully eliminated due to the reliance on cross-sectional, self-reported data. Future research should therefore employ multi-source and longitudinal designs to better mitigate potential method biases [61].Because the data is retrospective and self-reported, the validity of some measures in the study may be affected by potential recall biases [66]. The variance observed in the model might stem from the measurement method itself rather than the constructs being evaluated. This potential single-source bias could compromise the validity of the conclusions regarding the relationships between the measures [67]. Although this cross-sectional study does not enable strong assertions about temporal causality, it provides support for testing both a mediating effect, from a statistical viewpoint, that reveals potential mechanisms through which teacher self-efficacy and teacher collaboration influence the relationship between teacher innovation and teacher usage behavior of digital resources, as well as a moderating effect that highlights how teacher experience with professional development in digital resources may affect each pathway in the conceptual model [68,69]. A well-designed combination of future time-series, longitudinal, and detailed case study research would enhance this study by modeling and evaluating the dynamic and reciprocal relationships among relevant variables, offering deeper insights into teacher usage behavior of digital resources in teaching practice.

As outlined in the methods section, this article is built on an analysis of extensive, large-scale data using advanced statistical methods. Each construct in this article was assessed using a single scale. While the scales employed have been validated in prior literature, as outlined in earlier sections, it is important to acknowledge that they may not capture all dimensions of each construct. For instance, the construct of teacher innovation in this study was assessed based on teacher self-perceptions at the individual level and thus excluded the other domains of innovation like the collective level [70]. This article presents an opportunity for future research to conduct more comprehensive and in-depth measurements of each construct. Furthermore, this paper centers on exploring the mechanisms through which teacher innovation, teacher self-efficacy, and teacher collaboration influence teacher usage behavior of digital resources in teaching practice. Future research could explore the influence of additional potential factors, such as school leadership, digital infrastructure, learning climate [47], learning climate [12], and digital literacy [14] on teacher usage behavior of digital resources in teaching practice, using PISA datasets and beyond.

The scale and parameters of this article support the decision to concentrate on intricate analyses of the PISA data as a combined, decontextualized set across 81 nations. This method closely aligns with that of certain earlier articles [70,71]. (e.g., Lin & Gao, 2023; Nguyen et al., 2021). For example, Lin & Gao (2023) analyzed data from the TALIS 2018 survey, derived from 48 OECD countries, to investigate the factors that influence teacher autonomy in teaching [71]. This study expands on previous research by examining teacher use of digital resources in teaching across different geographical locations. Future studies could further enhance the understanding of this topic by analyzing OECD datasets to compare perspectives on this matter from both school and student viewpoints.

While subgroup or regional analyses could offer additional insights, the present study aimed to identify globally consistent mechanisms. Future research may employ multilevel or regional comparative models to further explore cross-national differences.

Finally, while descriptive statistics were weighted using the teacher sampling weights (W_TCHWT), the PLS-SEM analyses relied on unweighted data and did not model the multilevel structure of teachers nested within schools and countries. This limitation implies that standard errors and effect sizes may be somewhat biased, especially if selection probabilities and clustering are strongly related to the focal constructs. Future research should consider multilevel structural equation modelling or design-based approaches that can explicitly incorporate sampling weights and clustering when analyzing PISA teacher data.

### 6.5. Theoretical implications

This study extends innovation diffusion theory by integrating teacher collaboration as a systemic mediator, contrasting with prior individual-centric models.

### 6.6. Practical implications

Although some standardized path coefficients in this study are relatively small (β ≈ 0.10–0.20), such magnitudes are typical and meaningful in large-scale international education datasets like PISA. Small effects in large, heterogeneous samples often represent stable and generalizable relationships that hold across diverse contexts and education systems. In this study, the results reveal consistent global patterns linking teacher innovation, self-efficacy, collaboration, and digital resource usage. Therefore, despite modest statistical sizes, these associations hold substantial practical significance, highlighting critical leverage points for teacher training and educational digitalization policies. Policy makers should design professional development (PD) programs that pair technical training with collaborative innovation workshops.

## 7. Conclusion

Our research has contributed to understanding the relationships between teachers’ innovation and the usage behavior of digital resources. The findings confirm that teacher innovation positively impacts digital resource usage, with teacher self-efficacy and collaboration serving as mediators. Moreover, professional development moderates this relationship, reinforcing the need for targeted training. Practically, the study highlights the importance of fostering an innovative teaching culture and providing professional development opportunities to support digital integration. Policymakers should consider evidence-based strategies to promote teacher innovation, collaboration, and continuous learning for effective digital resource utilization. Future research should adopt a more holistic approach, integrating both internal and external factors to deepen theoretical and empirical insights into teachers’ digital resource usage.

## Supporting information

S1 File pisa_2022MS_overall_continuous. (XLSX)

## Abbreviations

TCL: Teacher Collaboration
TIN: Teacher Innovation
TSEF: Teacher self-efficacy
UBDR: Teacher usage behavior of digital resources
PD: for professional development

## References

[1] OECD. OECD Digital Education Outlook 2023: Towards an Effective Digital Education Ecosystem. OECD. 2023.

[2] Kearney M, Schuck S, Aubusson P, Burke PF. Teachers’ technology adoption and practices: lessons learned from the IWB phenomenon. Teacher Development. 2017;22(4):481–96. doi: 10.1080/13664530.2017.1363083

[3] Sailer M, Murböck J, Fischer F. Digital learning in schools: What does it take beyond digital technology?. Teaching and Teacher Education. 2021;103:103346. doi: 10.1016/j.tate.2021.103346

[4] Foster N. Teacher digital competences: formal approaches to their development. Paris: OCDE. 2023.

[5] Habibi A, Mukminin A, Hadisaputra P. Science teachers’ integration of digital resources in education: A survey in rural areas of one Indonesian province. Heliyon. 2020;6(8):e04631. doi: 10.1016/j.heliyon.2020.e04631 32793838 PMC7408340

[6] Hoareau L, Thomas A, Tazouti Y, Dinet J, Luxembourger C, Jarlégan A. Beliefs about digital technologies and teachers’ acceptance of an educational app for preschoolers. Comput Education. 2021;172:104264.

[7] Konstantinidou E, Scherer R. Teaching with technology: A large-scale, international, and multilevel study of the roles of teacher and school characteristics. Comput Education. 2022;179:104424. doi: 10.1016/j.compedu.2021.104424

[8] Akar SG, Mazman A. Does it matter being innovative: Teachers’ technology acceptance. Educ Inf Technol. 2019;24(6):3415–32.

[9] Paetsch J, Franz S, Wolter I. Changes in early career teachers’ technology use for teaching: the roles of teacher self-efficacy, ICT literacy, and experience during COVID-19 school closure. Teaching Teacher Educat. 2023;135:104318. doi: 10.1016/j.tate.2023.104318

[10] Sailer M, Stadler M, Schultz-Pernice F, Franke U, Schöffmann C, Paniotova V, et al. Technology-related teaching skills and attitudes: Validation of a scenario-based self-assessment instrument for teachers. Comput Human Behavior. 2021;115:106625. doi: 10.1016/j.chb.2020.106625

[11] Vidergor HE. The effect of teachers’ self- innovativeness on accountability, distance learning self-efficacy, and teaching practices. Comput Educat. 2023;199:104777. 10.1016/j.compedu.2023.104777 PMC9998282 36919161

[12] Wang J, Tigelaar DEH, Admiraal W. Rural teachers’ sharing of digital educational resources: From motivation to behavior. Comput Education. 2021;161:104055.

[13] Wu D, Yang X, Yang W, Lu C, Li M. Effects of teacher- and school-level ICT training on teachers’ use of digital educational resources in rural schools in China: A multilevel moderation model. Int J Educational Res. 2022;111:101910. doi: 10.1016/j.ijer.2021.101910

[14] Yao N, Wang Q. Factors influencing pre-service special education teachers’ intention toward AI in education: digital literacy, teacher self-efficacy, perceived ease of use, and perceived usefulness. Heliyon. 2024;10(14):e34894. doi: 10.1016/j.heliyon.2024.e34894 39149079 PMC11325385

[15] Drossel K, Eickelmann B, Gerick J. Predictors of teachers’ use of ICT in school – the relevance of school characteristics, teachers’ attitudes and teacher collaboration. Educ Inf Technol. 2017;22(2):551–73.

[16] Thurlings M, Evers AT, Vermeulen M. Toward a model of explaining teachers’ innovative behavior. Rev Educational Res. 2015;85(3):430–71. doi: 10.3102/0034654314557949

[17] Cai Y, Tang R. School support for teacher innovation: The role of basic psychological need satisfaction. Thinking Skills and Creativity. 2022;45:101096. doi: 10.1016/j.tsc.2022.101096

[18] Liu S, Yin H, Wang Y, Lu J. Teacher innovation: conceptualizations, methodologies, and theoretical framework. Teaching and Teacher Education. 2024;145:104611. doi: 10.1016/j.tate.2024.104611

[19] Sodergren CDC, Kettler T, Sulak T, Payne A. Teacher Self-Efficacy, Innovativeness, and Preparation to Teach Cross-Curriculum Skills. Int J of Cont Edu Res. 2023;10(1):197–209. doi: 10.33200/ijcer.1104747

[20] Vangrieken K, Dochy F, Raes E, Kyndt E. Teacher collaboration: a systematic review. Educational Res Rev. 2015;15:17–40. doi: 10.1016/j.edurev.2015.04.002

[21] Sawyer RK. The Cambridge handbook of the learning sciences. Cambridge University Press. 2005.

[22] Kelchtermans G. Teacher collaboration and collegiality as workplace conditions. A review. Zeitschrift für Pädagogik. 2006;52(2):220–37.

[23] Bovbjerg KM. Teams and collegiality in educational culture. European Educational Res J. 2006;5(3–4):244–53. doi: 10.2304/eerj.2006.5.3.244

[24] Datnow A. Collaboration and contrived collegiality: revisiting Hargreaves in the age of accountability. J Educ Change. 2011;12(2):147–58. doi: 10.1007/s10833-011-9154-1

[25] Visscher A, Witziers B. Subject departments as professional communities?. British Educational Res J. 2004;30(6):785–800. doi: 10.1080/0141192042000279503

[26] Meirink JA, Imants J, Meijer PC, Verloop N. Teacher learning and collaboration in innovative teams. Cambridge J Education. 2010;40(2):161–81. doi: 10.1080/0305764x.2010.481256

[27] García-Martínez I, Montenegro-Rueda M, Molina-Fernández E, Fernández-Batanero JM. Mapping teacher collaboration for school success. School Effect School Improvement. 2021;32(4):631–49. doi: 10.1080/09243453.2021.1925700

[28] Truijen KJP, Sleegers PJC, Meelissen MRM, Nieuwenhuis AFM. What makes teacher teams in a vocational education context effective?. J Workplace Learning. 2013;25(1):58–73. doi: 10.1108/13665621311288485

[29] Van Gasse R, Vanlommel K, Vanhoof J, Van Petegem P. The impact of collaboration on teachers’ individual data use. School Effectiveness and School Improvement. 2017;28(3):489–504. doi: 10.1080/09243453.2017.1321555

[30] Bandura A, Walters RH. Social learning theory. Englewood Cliffs, NJ: Prentice Hall. 1977.

[31] Tschannen-Moran M, Hoy AW. Teacher efficacy: capturing an elusive construct. Teaching and Teacher Education. 2001;17(7):783–805. doi: 10.1016/s0742-051x(01)00036-1

[32] Açikgül Firat E, Torun F. A Structural equation modelling of factors affecting the prospective teachers’ innovativeness level. Int J of Cont Edu Res. 2022;9(2):219–31. doi: 10.33200/ijcer.927884

[33] Postholm MB. Teachers’ professional development: a theoretical review. Educational Res. 2012;54(4):405–29. doi: 10.1080/00131881.2012.734725

[34] Baporikar N. Understanding professional development for educators. International J Sustainable Economies Management. 2015;4(4):18–30. doi: 10.4018/ijsem.2015100102

[35] Fullan M. Successful school improvement: The implementation perspective and beyond. McGraw-Hill Education (UK). 1992.

[36] Rehman N, Huang X, Mahmood A, Abbasi MS, Qin J, Wu W. Assessing Pakistan’s readiness for STEM education: an analysis of teacher preparedness, policy frameworks, and resource availability. Humanit Soc Sci Commun. 2025;12(1). doi: 10.1057/s41599-025-05584-3

[37] Mahmood A, Rehman N, Huang X, Zamani N. Effect of strategic memory advanced reasoning training (SMART) therapy for enhancing final-year high school students career choices. BMC Psychol. 2025;13(1):445. doi: 10.1186/s40359-025-02767-0 40296108 PMC12036163

[38] Peeraer J, Van Petegem P. The limits of programmed professional development on integration of information and communication technology in education. AJET. 2012;28(6). doi: 10.14742/ajet.809

[39] Fernández-Batanero JM, Montenegro-Rueda M, Fernández-Cerero J, García-Martínez I. Digital competences for teacher professional development. systematic review. European J Teacher Education. 2020;45(4):513–31. doi: 10.1080/02619768.2020.1827389

[40] Xie K, Nelson MJ, Cheng SL, Jiang Z. Examining changes in teachers’ perceptions of external and internal barriers in their integration of educational digital resources in K-12 classrooms. J Res Technol Education. 2023;55(2):281–306.

[41] Xie K, Di Tosto G, Chen SB, Vongkulluksn VW. A systematic review of design and technology components of educational digital resources. Computers & Education. 2018;127:90–106.

[42] Kim C, Kim MK, Lee C, Spector JM, DeMeester K. Teacher beliefs and technology integration. Teaching and Teacher Education. 2013;29:76–85. doi: 10.1016/j.tate.2012.08.005

[43] Davis FD. Technology acceptance model: TAM. Information Seeking Behavior and Technol Adoption. 1989;:205–19.

[44] Wang J, Tigelaar DEH, Admiraal W. Connecting rural schools to quality education: Rural teachers’ use of digital educational resources. Computers in Human Behavior. 2019;101:68–76. doi: 10.1016/j.chb.2019.07.009

[45] Yu J, Vidal Q, Vincent-Lancrin S. Digital teaching and learning resources. Paris: OECD. 2023.

[46] Alkawsi G, Ali N, Baashar Y. The moderating role of personal innovativeness and users experience in accepting the smart meter technology. Applied Sciences. 2021;11(8):3297. doi: 10.3390/app11083297

[47] Vermeulen M, Kreijns K, van Buuren H, Van Acker F. The role of transformative leadership, ICT‐infrastructure and learning climate in teachers’ use of digital learning materials during their classes. Brit J Educational Tech. 2016;48(6):1427–40. doi: 10.1111/bjet.12478

[48] Behavioral change models. The Compass for SBC. 2023.

[49] Rogers EM, Singhal A, Quinlan MM. Diffusion of innovations. An integrated approach to communication theory and research. Routledge. 2014. p. 432–48.

[50] OECD. PISA 2022 Assessment and Analytical Framework. Paris: Organisation for Economic Co-operation and Development. 2023.

[51] Kock N. Common method bias in PLS-SEM: A full collinearity assessment approach. Int J e-Collaboration. 2015;11(4):1–10.

[52] Petter S, Straub D, Rai A. Specifying Formative Constructs in Information Systems Research1. MIS Quarterly. 2007;31(4):623–56. doi: 10.2307/25148814

[53] Barclay D, Higgins C, Thompson R. The partial least squares (PLS) approach to casual modeling: personal computer adoption and use as an illustration. 1995.

[54] Chin WW. The partial least squares approach to structural equation modeling. Modern methods for business research. 1998;295(2):295–336.

[55] Hulland J. Use of partial least squares (PLS) in strategic management research: a review of four recent studies. Strat Mgmt J. 1999;20(2):195–204.

[56] Fornell C, Larcker DF. Evaluating structural equation models with unobservable variables and measurement error. J Marketing Res. 1981;18(1):39. doi: 10.2307/3151312

[57] Henseler J, Ringle CM, Sarstedt M. A new criterion for assessing discriminant validity in variance-based structural equation modeling. J Acad Mark Sci. 2015;43(1):115–35.

[58] Chin WW. The partial least squares approach to structural equation modeling. Modern Methods Business Res. 1998;295(2):295–336.

[59] Wetzels M, Odekerken-Schröder G, van Oppen C. Using PLS path modeling for assessing hierarchical construct models: guidelines and empirical illustration1. MIS Quarterly. 2009;33(1):177–95. doi: 10.2307/20650284

[60] Henseler J, Ringle CM, Sinkovics RR. The use of partial least squares path modeling in international marketing. New challenges to international marketing. Emerald Group Publishing Limited. 2009. p. 277–319.

[61] Podsakoff PM, MacKenzie SB, Lee J-Y, Podsakoff NP. Common method biases in behavioral research: a critical review of the literature and recommended remedies. J Appl Psychol. 2003;88(5):879–903. doi: 10.1037/0021-9010.88.5.879 14516251

[62] Hair JF. A primer on partial least squares structural equation modeling (PLS-SEM). Second ed. Los Angeles: Sage. 2017.

[63] Baron RM, Kenny DA. The moderator-mediator variable distinction in social psychological research: conceptual, strategic, and statistical considerations. J Pers Soc Psychol. 1986;51(6):1173–82. doi: 10.1037//0022-3514.51.6.1173 3806354

[64] Chin WW. How to Write Up and Report PLS Analyses. In: Esposito Vinzi V, Chin WW, Henseler J, Wang H, editors. Handbook of Partial Least Squares. Berlin, Heidelberg: Springer Berlin Heidelberg; 2010. p. 655–90.

[65] Çelik K. The relationship between individual innovativeness and self-efficacy levels of student teachers. Int J Scientific Res Educ. 2013;6(1):56–67.

[66] Schwarz N. Retrospective and concurrent self-reports: The rationale for real-time data capture. Science Of Real-time Data Capture: Self-reports In Health Research. 2007. p. 26.

[67] Podsakoff PM, MacKenzie SB, Lee J-Y, Podsakoff NP. Common method biases in behavioral research: a critical review of the literature and recommended remedies. J Appl Psychol. 2003;88(5):879–903. doi: 10.1037/0021-9010.88.5.879 14516251

[68] Baron RM, Kenny DA. The moderator-mediator variable distinction in social psychological research: conceptual, strategic, and statistical considerations. J Pers Soc Psychol. 1986;51(6):1173–82. doi: 10.1037//0022-3514.51.6.1173 3806354

[69] Cautin RL, Lilienfeld SO. The Encyclopedia of Clinical Psychology, 5 Volume Set. John Wiley & Sons. 2015.

[70] Nguyen D, Pietsch M, Gümüş S. Collective teacher innovativeness in 48 countries: Effects of teacher autonomy, collaborative culture, and professional learning. Teaching Teacher Education. 2021;106:103463. doi: 10.1016/j.tate.2021.103463

[71] Lin Q, Gao X. Exploring the predictors of teachers’ teaching autonomy: A three-level international study. Teaching and Teacher Education. 2023;135:104338. doi: 10.1016/j.tate.2023.104338

